# The Association of the Low-Income Housing Tax Credit Program and Intimate Partner Violence Related Emergency Department Visits

**DOI:** 10.1007/s10896-024-00750-8

**Published:** 2024-09-28

**Authors:** Meghan E. Shanahan, Anna E. Austin, Christine P. Durrance, Sandra L. Martin, Desmond K. Runyan, Carol W. Runyan

**Affiliations:** 1 Department of Maternal and Child Health, Gillings School of Global Public Health, University of North Carolina at Chapel Hill, 421 Pittsboro St, CB# 7445, Chapel Hill, NC 27599-7445 USA; 2 Injury Prevention Research Center, University of North Carolina at Chapel Hill, Chapel Hill, USA; 3 Department of Health Behavior, Gillings School of Global Public Health, University of North Carolina at Chapel Hill, Chapel Hill, USA; 4 LaFollette School of Public Affairs, University of Wisconsin-Madison, Madison, USA; 5 Department of Pediatrics, School of Medicine, University of North Carolina at Chapel Hill, Chapel Hill, USA

**Keywords:** Intimate Partner Violence, Affordable Housing, Social Determinants of Health, Prevention

## Abstract

**Purpose:**

To examine the association of increasing access to affordable housing through the Low-income Housing Tax Credit Program with non-fatal intimate partner violence (IPV) among women.

**Methods:**

We used 2005–2014 data from the State Emergency Department Database (SEDD) and the low-income housing tax credit (LIHTC) program, a federal program providing tax incentives to support the construction of affordable housing units, to examine the association between the number of LIHTC units available and rates of IPV-related emergency department visits. We conducted generalized linear regression comparing state-years with above the median number of LIHTC units (> 28 units per 100,000 population) to those with below the median number of units (≤ 28 LIHTC units per 100,000 population). We adjusted the analyses for time-varying indicators of state median household income, percent population by race/ethnicity, percent population unemployed, violent crime rate, refundable Earned Income Tax Credits, maximum Temporary Assistance for Needy Families benefit for family of 3, and minimum wage.

**Results:**

We found that greater availability of LIHTC units (> 28 vs. ≤ 28 LIHTC units per 100,000 population) was associated with decreased rates of IPV-related emergency department visits among women (RR 0.90; 95% CI 0.84, 0.97).

**Conclusions:**

Increasing access to affordable housing, an important social determinant of health, through the LIHTC program may be an important component of strategies to prevent IPV severe enough to warrant an ED visit.

**Supplementary Information:**

The online version contains supplementary material available at 10.1007/s10896-024-00750-8.

## Introduction

Intimate partner violence (IPV), which includes physical and sexual violence, stalking, and psychological aggression (Centers for Disease Control & Prevention, 2022), is a critical public health issue affecting 47% of women in their lifetime (Leemis et al., 2022). While both women and men are at risk of IPV, women are more likely to experience IPV compared to men (Centers for Disease Control & Prevention, 2022). In addition to IPV-related injuries, individuals who experience IPV are at an increased risk of engaging in negative health behaviors, such as binge drinking and decreased preventative care utilization, as well as an increased risk of developing chronic physical and mental health conditions (Black, 2011; Breiding et al., 2008). These consequences result in substantial economic costs. Recent estimates indicate that IPV costs $103,767 per female victim and the lifetime population-level economic burden of IPV is $3.6 trillion (Peterson et al., 2018).

Numerous risk factors for IPV have been identified, including attitudes accepting of violence, unhealthy family interactions, high rates of violence and crime in communities, and the presence of social norms regarding gender inequality (Capaldi et al., 2012; Centers for Disease Control & Prevention, 2022; Schwab-Reese et al., 2016; Stith et al., 2004). Economic stress is a risk factor that affects IPV among individuals, families, and communities (Centers for Disease Control & Prevention, 2021; Khalifeh et al., 2013; Matjasko et al., 2013). Given that the cost of housing, whether it be paying for mortgage or rent, has been outpacing average incomes (Hermann, 2018; Schuetz, 2019), it is likely that increased housing cost is a significant contributor to economic stress. Relatedly, access to safe, affordable housing is a protective factor for IPV (Centers for Disease Control & Prevention, 2021) by decreasing stress associated with housing cost and instability and by helping to facilitate exits from violent relationships. Therefore, it is possible that increasing the availability of affordable housing could reduce rates of IPV.

Indeed, previous research indicates that addressing housing insecurity among women who experience IPV is an effective tertiary prevention strategy (Sullivan & Olsen, 2016; Yakubovich et al., 2022), increasing safety and improving mental health outcomes (Yakubovich et al., 2022). Typically, domestic violence shelters and transitional housing are used to address housing instability among women experiencing IPV (Fraga Rizo et al., 2022). However, as these services are inherently temporary solutions and risk factors for IPV are often intractable and therefore may take time to ameliorate (World Health Organization & Pan American Health Organization, 2012), there is a need to consider additional strategies that may provide long-term, ongoing safe and stable housing to those experiencing IPV. Additionally, improving access to affordable housing could reduce stress in intimate relationships, thereby reducing IPV (Benson et al., 2003). One potential durable solution is to expand access to affordable housing through the Low-Income Housing Tax Credit Program (LIHTC), the largest federal program to provide affordable housing in the US (US Department of Housing and Urban Development, n.d.). This program provides approximately 2.7 million affordable housing units across the country (US Department of Housing and Urban Development, 2019). Created through the 1986 Tax Reform Act, the goal of the LIHTC program is to incentivize the development of low-income housing units by providing tax credits to housing developers that prioritize providing longer-term affordable housing to low-income individuals. Previous research has demonstrated that affordable housing provided through the LIHTC program is associated with reductions in violent crime (Freedman & Owens, 2011). Given the focal population of the LIHTC program, and that being low-income is a strong predictor of IPV, it is likely that increasing access to affordable housing through LIHTC program could reduce rates of IPV (Capaldi et al., 2012). Indeed, previous work has demonstrated that increasing access to affordable housing through the LIHTC program is associated with reduced rates of IPV-related homicides (Austin et al., 2022). It is not known, however, if increasing access to affordable housing through the LIHTC program prevents more common, non-fatal IPV incidents.

A recent study that matched perpetrators of IPV-homicide to perpetrators of non-fatal IPV found that perpetrators of IPV-homicide were more likely to have previously perpetrated IPV than perpetrators of non-fatal IPV (Jung & Stewart, 2019). This indicates that IPV homicide is an escalation of IPV behaviors and therefore, preventing non-fatal IPV may be an opportunity to stop violent behavior before it escalates in severity. Further, given that increased access to affordable housing prevents fatal IPV (Austin et al., 2022), it is also possible that it is also an effective prevention strategy for non-fatal IPV.

In the current study, we examine the association between the availability of LIHTC units and emergency department (ED) visits for injuries related to IPV against women at the state-level. While many individuals who experience IPV do not seek medical care, among those who do, many initiate health care through the ED (Singhal et al., 2021), therefore this is an effective way to capture non-fatal incidents of IPV.

## Methods

We used two data sources to examine the association between the number of LIHTC units available and IPV-related emergency department visits at the state-level between 2005 and 2014: 1) 2005–2014 data from the LIHTC Database from the Department of Housing and Urban Development and 2) 2005–2014 data from the Healthcare Cost and Utilization Project’s (HCUP) State Emergency Department Database (SEDD). The SEDD includes visits to hospital owned emergency departments that do not result in hospital admissions. We used data from 13 states: Arizona, Florida, Hawaii, Iowa, Massachusetts, Maryland, Maine, North Carolina, Nebraska, New Jersey, New York, Rhode Island, and Utah. We chose these states based on completeness of SEDD data from 2005–2014 (i.e., states with consistent data for the years under study as indicated by the HCUP Database Catalog) as well as budget constraints in obtaining the data. The SEDD is a primary source of data for studying injuries and nonfatal, preventable events (Hirshon et al., 2009).

### Measures

*LIHTC Units*. Our exposure was the number of LIHTC units per 100,000 population by state and year. We used the year the LIHTC units were placed in service rather than the year the tax credit was allocated since the date placed in service represents when the unit was available for rent. As there is no objective measure of LIHTC unit availability, we used the median number of LIHTC units per 100,000 population. Since the median number of LIHTC units per 100,000 population was 28, we identified each state-year as having > 28 or ≤ 28 LIHTC units per 100,000 population. Population estimates included all individuals in the US and were obtained from the National Center for Health Statistics.

*IPV-related Emergency Department Visits.* We counted any emergency department visit with any of the ICD-9-CM diagnosis codes included 995.80, 995.81, 995.82, 995.85, or E967.3 (Btoush et al., 2008; Table 1). While this definition is based in previous literature (Btoush et al., 2008), it is slightly more conservative as it does not include adult sexual abuse, observation for abuse, marital problems, history of physical abuse, and history of emotional abuse.

**Table 1:** ICD-9-CM Codes for IPV-related ED visits

ICD-9-CM Billing Code	
995.80	Adult maltreatment NOS
995.81	Adult physical abuse
995.82	Adult psychological abuse
995.85	Other adult abuse and neglect
E967.3	Perpetrator of child and adult abuse, by spouse or partner

*Sex.* We used an indicator of biological sex to identify IPV against women.

*Confounders.* We used existing literature, theory, and our subject matter expertise to create a directed acyclic graph (DAG) to identify potential confounders of the relationship between LIHTC units and IPV related emergency department visits. Based on this DAG, we included the following time-varying indicators in our model: state median household income, percent population by race/ethnicity, unemployment rate, violent crime rate, refundable state Earned Income Tax Credits, maximum Temporary Assistance for Needy Families (TANF) benefit for family of 3, and state minimum wage.

### Analysis

We conducted generalized linear regression with generalized estimating equations (GEE) to account for repeated measures within states over time (Hanley et al., 2003), using an exchangeable correlation matrix and robust variance estimators:

$${Y}_{st}={\beta }_{0}+{\beta }_{1}{LIHTC}_{st}+{\beta }_{2}{X}_{st}+{\beta }_{3}{Year}_{t}+log\left({Population}_{st}\right)+{e}_{st}$$

where *s* indicates state and *t* indicates year. The outcome *Y*_*st*_ is the number of IPV emergency department visits in state *s* and year *t*. *LIHTC*_*st*_ is a binary variable indicating whether the number of LIHTC units placed in service per 100,000 population in state *s* and year *t* is > 28 or ≤ 28 units, and *β*_*1*_ represents the association of LIHTC unit availability with IPV. *X*_*st*_ represents the confounders described above. *Year*_*t*_ is an indicator for year to account for secular trends over time. *Population*_*st*_ is the total population in state *s* and year *t*, with log(*Population*_*st*_) included in the model to account for differences in population size by state and year. As each state contributed multiple years to the analysis, we used state-years as the unit of analysis. We used a log link and negative binomial distribution to calculate rate ratios (RRs) and 95% confidence intervals (CIs) comparing the rate of IPV emergency department visits in state-years with > 28 to state-years with ≤ 28 LIHTC units per 100,000 population.

We conducted analyses in SAS 9.4. In line with guidance from the American Statistical Association, we focus on the magnitude of the point estimate for the RR and the width and location of the corresponding 95% CI when interpreting our results (Wasserstein & Lazar, 2016). The study was reviewed and considered exempt by the Institutional Review Board at the [redacted].

## Results

From 2005–2014 there were a total of 123 state-years included in the analysis. State-years with > 28 LIHTC units per 100,000 population differed from state-years with ≤ 28 LIHTC units per 100,000 population on several key demographic characteristics (Table 2). The median percent of the population that was Black, non-Hispanic was higher (7.0%) in state-years with > 28 LIHTC units per 100,000 population compared to the median percent that was Black, non-Hispanic (4.9%) in state-years with ≤ 28 LIHTC units per 100,000 population. Additionally, state-years with > 28 LIHTC units per 100,000 population had a larger median Hispanic population (11%), higher median violent crime rate (355 per 100,000 population), and a higher median maximum TANF benefit for a family of three ($482) compared to state-years with ≤ 28 LIHTC units per 100,000 population (9.7%, 296.6 per 100,000 population, and $450, respectively).

**Table 2:** Demographic characteristics of state-years with ≤ 28 LIHTC and > 28 LIHTC units per 100,000 population

	≤ 28 LIHTC units per 100,000 population (*N* = 60)		> 28 LIHTC units per 100,000 population (*N* = 63)	
	Mean	Median	Mean	Median
Median household income	$54,673	$51,256	$55,328	$54,562
Percent population White, non-Hispanic	68.6	66.7	67.9	62.7
Percent population Black, non-Hispanic	8.1	4.9	11.0	7.0
Percent population other race, non-Hispanic	10.5	4.3	8.6	5.2
Percent population Hispanic	12.8	9.7	12.4	11.0
Percent population unemployed	6.7	6.7	5.9	5.2
Violent crime rate per 100,000 population	323.5	296.6	385.2	355.0
State minimum wage	7.18	7.25	6.77	7.25
Maximum TANF benefit for family of 3	$448	$450	$498	$482
IPV ED visits among women per 100,000 population	21.7	20.9	21.3	20.7

LIHTC and SEDD data, 2005–2014. *N* = 123 state/year observations. Maximum *N* = 123 total state-years from 2005–2014 (Arizona, Florida, Hawaii, Iowa, Massachusetts, Maryland, Maine, North Carolina, Nebraska, New Jersey, New York, Rhode Island, and Utah). Binary indicators (e.g., refundable state Earned Income Tax Credit) included in our models are suppressed from this table.

In unadjusted models, we found that the rate of IPV-related emergency department visits among women in state-years with > 28 LIHTC units per 100,000 population was 0.96 (95% CI 0.88, 1.05) times that of the rate of ED IPV visits among women in state-years with ≤ 28 LIHTC units per 100,000 population (Table 3). When adjusting for potential confounders, we found that greater availability of LIHTC units (> 28 vs. ≤ 28 LIHTC units per 100,000 population) was associated with decreased rates of IPV-related emergency department visits among women (RR 0.90; 95% CI 0.84, 0.97; Table 3).

**Table 3:** Association between availability of LIHTC units per 100,000 population and ED visits for IPV-related injuries among women

	Unadjusted RR (95% CI)	Adjusted RR (95% CI)
IPV ED visits among women	0.96 (0.88, 1.05)	0.90 (0.84, 0.97)

LIHTC units per 100,000 population and offset term for the total population. Maximum *N* = 123 total state-years from 2005–2014 (Arizona, Florida, Hawaii, Iowa, Massachusetts, Maryland, Maine, North Carolina, Nebraska, New Jersey, New York, Rhode Island, and Utah). Adjusted RR includes median household income, percent population by race/ethnicity, unemployment rate, violent crime rate, maximum TANF benefit for family of 3, refundable state Earned Income Tax Credit, and state minimum wage.

## Discussion

As the largest federal program to create affordable housing in the US, the LIHTC program plays an important role in ensuring the well-being and safety of women. In this study, we found that state-years with > 28 LIHTC units per 100,000 population had lower rates of emergency department visits related to IPV than state-years with ≤ 28 LIHTC units per 100,000 population. We conducted sensitivity analysis using quartiles of LIHTC rates per state-year instead of the median rate of LIHTC units per state-year and found similar results (Supplement). These results suggest that expanding access to affordable housing, an important social determinant of health (HHS, Office of Disease Prevention and Health Promotion, n.d.), is a potential strategy to reduce IPV in communities.

Our results add to the growing body of literature demonstrating the importance of housing for women who experience or who are at-risk of experiencing IPV. As previously mentioned, increased availability of LIHTC units has been found to be associated with reduced rates of IPV-related homicides (Austin et al., 2022). Our results indicate that increasing access to affordable housing through the LIHTC program may also be an effective component of strategies to reduce non-fatal IPV. Additionally, while it is well-established that women who experience IPV benefit from domestic violence shelters, transitional housing, and housing vouchers (Klein et al., 2021), our findings suggest that addressing housing insecurity by increasing the supply of affordable rental housing may also be a potential element of comprehensive strategies to reduce IPV against women.

There are several potential mechanisms that could explain these findings. Previous research has found that experiencing financial stress increases the odds of IPV (Schwab-Reese et al., 2016). Specifically, as the number of financial stressors (e.g., utilities nonpayment, housing nonpayment, food insecurity) increased, the odds of severe physical IPV increased 1.22 times (95% CI 1.14, 1.30) and the odds of IPV resulting in injury increased 1.27 times (95% CI 1.16, 1.38). Housing stress specifically, as measured by housing nonpayment, increased the odds of severe physical IPV by 1.74 (95% CI 1.74, 2.71) and the odds of IPV resulting in injury by 1.78 (95% CI 1.21, 2.59) (Schwab-Reese et al., 2016). These results suggest that financial stress broadly, and housing stress specifically, may precipitate incidents of IPV. Therefore, increasing access to affordable housing may reduce these stressors and consequently reduce rates of IPV-related injuries. Further, previous qualitative research exploring the housing needs of women who have experienced IPV found that increasing access to affordable and safe housing is necessary to improve safety for women and their children (Clough et al., 2014). Women in the study recounted not being able to afford rent or mortgage payments without their partner’s earnings, thereby limiting their ability to leave an abusive situation. Increasing the supply of affordable housing units could make it more likely that some women could cover housing payments without their partner, thus increasing their ability to remove themselves from relationships where violence is likely to occur.

Our results align with other research demonstrating that economic support programs are associated with reductions in IPV. Previous work has demonstrated that the refundable EITC was associated with reductions in coercive IPV, particularly isolation and financial coercion (Spencer et al., 2020). Additional work found that increasing the EITC benefit based on family size under OBRA-93 reduced the prevalence of IPV (Cesur et al., 2022). Further work should examine additional economic support programs and policies, such as increases in the Federal minimum wage and Medicaid expansion, on rates of IPV.

### Limitations

This study has several limitations. The data included in this analysis are at the ecological level, therefore, we are not able to determine whether women who experienced IPV-related injuries were living in LIHTC units. We included 13 states in the analysis, and therefore, the results may not generalize to states not included in the study. While we used a comprehensive DAG to identify state-level characteristics and policies that may confound the association between availability of LIHTC units and IPV-related injuries treated in the emergency department, there is the possibility of unmeasured confounding due to factors such as median rent or other characteristics of the housing market. Given that LIHTC units are often placed in areas with higher levels of poverty, and poverty is associated with an increased risk of IPV, not being able to fully account for this endogeneity could bias our results away from the null.

Since the SEDD data only include emergency department visits that did not result in a hospital admission, we likely did not include the most severe cases of IPV injuries in this study, as those would likely be admitted to the hospital. On the other hand, our measures do not capture all IPV against women, as many IPV incidents are not treated in an emergency department or identified as IPV by the ICD-9-CM codes we used. Although unlikely, it is also possible that the codes we chose to identify IPV-related ED visits were applied to visits where IPV did not occur. Additionally, given the transition from ICD-9-CM to ICD-10-CM in 2015, we were not able to include data after 2014, as differences in rates of IPV-related ED visits when comparing across this transition period could be due to differences in coding, rather than due to the availability of LIHTC units. Further research should explore the association between increasing access to affordable housing and rates of IPV in more recent data.

## Conclusions

This study adds to the growing body of literature demonstrating that addressing social determinants of health may offer an effective strategy to reduce violence. The results of this study provide evidence that increasing access to affordable housing, an important social determinant of health, through the LIHTC program is associated with reduced rates of IPV related ED visits. These results have important implications for state and federal policies regarding increasing the supply of safe and affordable housing in communities. In supporting housing programs like LIHTC, policy makers should think broadly about their potential to support not only individual basic needs but also health and well-being.

## Supplementary Information

Below is the link to the electronic supplementary material.

Supplementary file1 (DOCX 14.8 KB)

## Data Availability

As this was a secondary data analysis and the data were accessed under a data use agreement with HCUP, a data availability statement is not applicable.
