# Supplementary material for: The Association of the Low-Income Housing Tax Credit Program and Intimate Partner Violence Related Emergency Department Visits
Source: J Fam Violence. 2024 Sep 28;41(1):187–93. doi: 10.1007/s10896-024-00750-8 (PMC12831692; doi:10.1007/s10896-024-00750-8)
Supplement: Supplementary file 1 — Supplementary file1 (DOCX 14.8 KB) [file 10896_2024_750_MOESM1_ESM.docx]

**Supplement**

Table 1. Association between availability of LIHTC units per 100,000 population (quartiles) and ED visits for IPV-related injuries among women.

| **IPV-related ED visits among women** | **Unadjusted RR**  **(95% CI)** | **Adjusted RR**  **(95% CI)** |
| --- | --- | --- |
| Quartile 2 vs Quartile 1 | 0.97 (0.91, 1.03) | 0.99 (0.93, 1.05) |
| Quartile 3 vs Quartile 1 | 0.95 (0.85, 1.05) | 0.90 (0.83, 0.99) |
| Quartile 4 vs Quartile 1 | 0.95 (0.88, 1.04) | 0.89 (0.83, 0.96) |

Notes: LIHTC units per 100,000 population and offset term for the total population. Maximum N=123 total state-years from 2005-2014 (Arizona, Florida, Hawaii, Iowa, Massachusetts, Maryland, Maine, North Carolina, Nebraska, New Jersey, New York, Rhode Island, and Utah). Adjusted RR includes median household income, percent population by race/ethnicity, unemployment rate, violent crime rate, maximum TANF benefit for family of 3, refundable state Earned Income Tax Credit, and state minimum wage.
